# Bilateral Facial Artery Hypoplasia in the Face: Surgical and Aesthetic Implications

**DOI:** 10.7759/cureus.106597

**Published:** 2026-04-07

**Authors:** Kaden L Wilson, Peyton Mraz, Jay M Bauman

**Affiliations:** 1 Anatomical Sciences, Saint Louis University School of Medicine, Saint Louis, USA

**Keywords:** angular artery, bilateral asymmetry, cadaveric dissection, facial artery, pre-operative imaging

## Abstract

While individual variations in the branching pattern of the facial artery have been reported, the simultaneous absence of multiple major branches is exceedingly rare. During routine cadaveric dissection of the superficial face, a unique combination of facial artery terminal branches was observed in an 85-year-old man. On the left side, the inferior labial artery and angular artery were absent. On the right side, the superior labial artery was absent. Additionally, the transverse facial artery was absent bilaterally. Consequently, the dorsal nasal artery and infraorbital arteries provided supplemental blood supply to the face, which is underreported in the literature. This combination of variations could significantly impact aesthetic procedures and head and neck surgeries, underscoring the need for awareness of these variations in preoperative and postoperative planning.

## Introduction

The arterial blood supply of the face is known to be highly variable, with many different classification systems. This can present significant challenges for surgeons and aesthetic practitioners. The facial artery (FA) is the principal blood supply to the face. In its classical configuration, the FA arises from the external carotid artery (ECA) just superior to the lingual artery and follows a tortuous course in the face to accommodate movements associated with mastication and facial expression [1].

As the FA courses superiorly along the face, it gives rise to several clinically important branches. After emerging from the inferior border of the mandible, the FA may give off a premasseteric branch (PMB) to supply the anterior masseter muscle [2]. The FA then ascends medially toward the oral commissure, branching into the inferior labial artery (ILA), which supplies the oral mucosa, the inferior portion of the orbicularis oris, and the inferior labial glands. The superior labial artery (SLA) subsequently arises to supply the upper lip, superior orbicularis oris, and nasal septum. These labial arteries typically anastomose with their contralateral counterparts.

The FA then branches as the lateral nasal artery (LNA), which supplies tissue near the ala of the nose, after which the FA terminates as the angular artery (AA). The AA travels along the lateral aspect of the nasal dorsum and forms a rich anastomotic network with vessels near the medial canthus.

In addition to the FA, other arteries contribute to the facial blood supply. The transverse facial artery (TFA), a branch of the superficial temporal artery, courses horizontally between the zygomatic arch and parotid duct and may form multiple anastomoses with anterior facial vessels [3]. The dorsal nasal artery (DNA), a terminal branch of the ophthalmic artery (OA) from the internal carotid artery (ICA), supplies the external superior nose. The infraorbital artery (IOA), a branch of the maxillary artery, emerges through the infraorbital foramen to supply the lower eyelid and midface structures.

Variations in FA anatomy are well documented, though classification systems differ considerably in nomenclature, criteria for inclusion, and anatomical landmarks [4-6]. Among the most clinically significant variations are early terminations of the FA. In most cases, the TFA provides compensatory circulation [7]. However, when the TFA is also absent, the face must rely on alternative collateral pathways, placing greater dependence on deep anatomic routes and increasing the risk of complications during maxillofacial surgery, filler injection, and flap procedures.

In this report, we describe a rare bilateral asymmetric FA termination pattern characterized by combined branch absences on both sides of the face, bilateral absence of the TFA, and compensatory flow from the IOA and DNA. This combination of findings has not been previously reported and is not well represented by existing classification frameworks.

## Case presentation

A rare anatomic variation was discovered during a routine medical school anatomy dissection course. The body donor was an 85-year-old male who passed away from chronic obstructive pulmonary disease, renal carcinoma, and dementia. All donors were obtained through the Saint Louis University Gift Body Program of the Center for Anatomical Science and Education (CASE) with signed, informed consent from the donors. All rules set forth by the Uniform Anatomical Gift Act are followed by the CASE Gift Body Program. No other anatomical anomalies or variations were found. 

**Figure 1:**
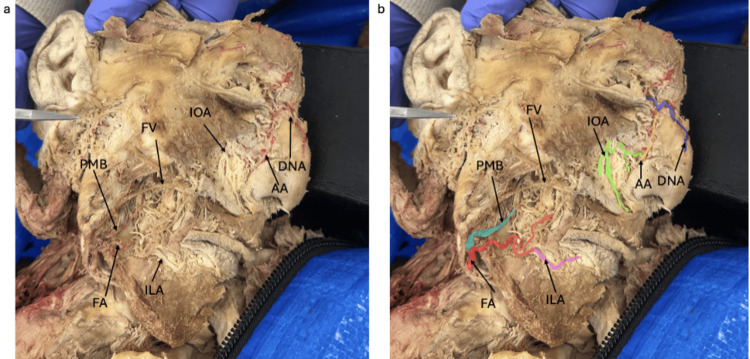
Right-Sided Early Termination Dissection demonstrating early termination of the facial artery on the right hemiface (a) with color-coded highlights (b). FA facial artery (red), PMB premasseteric branch (light blue), ILA inferior labial artery (purple), IOA infraorbital artery (green), AA angular artery (yellow), DNA dorsal nasal artery (blue), FV facial vein

Upon dissection, the terminal branches of the FA were found to be bilaterally asymmetric. On the right side, the FA branched from the ECA in its expected location (Figure 1). It gave off a PMB, which coursed superiorly to supply the anterior portion of the masseter muscle. The FA then continued superiorly and medially, terminating as a large ILA that coursed toward the midline to supply the lower lip. The right FA also contributed small muscular branches traveling superiorly that were lateral to the upper lip. These small branches pierced the buccinator muscle. No SLA was identified on the right side.

**Figure 2:**
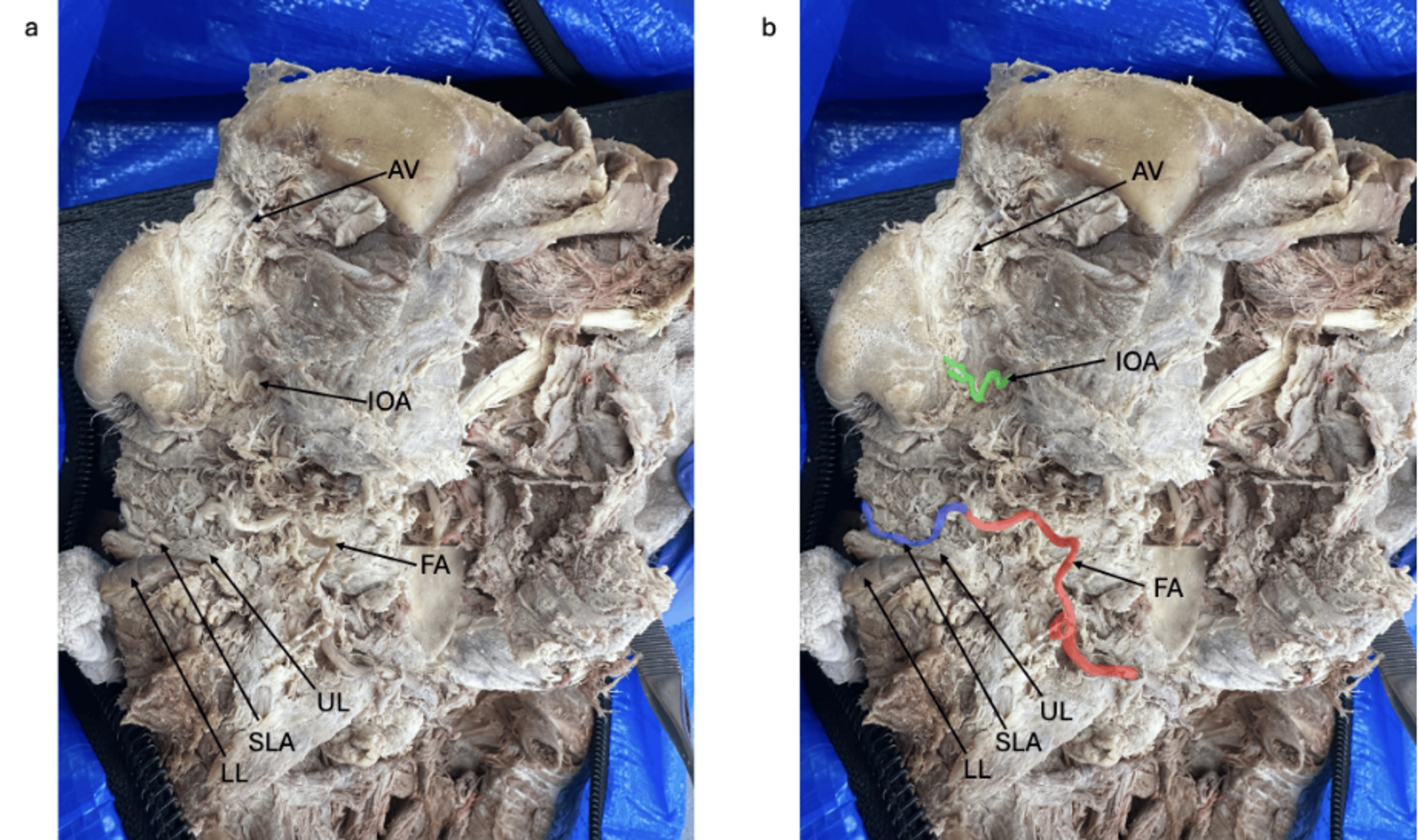
Left-Sided Early Termination Dissection demonstrating early termination of the facial artery on the left hemiface (a) with color-coded highlights (b). FA facial artery (red), SLA superior labial artery (blue), IOA infraorbital artery (green), AV angular vein, UL upper lip, LL lower lip

On the left side, the FA also arose from the ECA in its normal position (Figure 2). No PMB was observed. As dissection progressed, it was determined that the ILA was absent. The left FA changed course medially toward the oral commissure, where it continued as a hyperplastic SLA. This enlarged SLA coursed to the midline, supplying the upper lip, and represented the terminal branch of the left FA.

Further investigation revealed asymmetry involving the angular arteries. The right AA was present in its expected anatomical position lateral to the nasal dorsum. However, it was not a branch of the FA. Instead, the right AA was supplied primarily via an anastomosis with the right DNA, a branch of the OA. On the left side, the AA was entirely absent. A prominent angular vein was observed on the left, but no corresponding artery was identified.

Regarding compensatory blood flow, the TFA was absent bilaterally. The right IOA demonstrated multiple branches coursing inferiorly and medially to supply the skin, muscles, and mucosa of the superior lip and contributed to the right AA through a small anastomosis. The left IOA branched medially toward the lateral nasal dorsum, piercing the skin at the superior nasolabial fold to supply underlying structures.

## Discussion

The present case demonstrated an undescribed bilateral FA configuration in which the right FA terminated as the ILA (with absent SLA), and the left FA terminated as a hyperplastic SLA (with absent ILA and AA), accompanied by bilateral TFA absence and compensatory supply from the IOA and DNA. This combination of findings is not adequately captured by existing classification systems.

Across the literature, there is substantial variation in reported FA termination patterns, as well as in the classification schemes used to describe them. This lack of uniformity complicates comparisons across studies and limits clear interpretation of FA termination data. For example, Hong et al. [4] group the alar branches and LNA together when classifying terminal vessels, whereas others distinguish between them as separate termination points. Loukas et al. [5] identify the SLA and ILA as distinct endpoints, while Wang et al. [6] group them together.

Despite ambiguity in the literature, the angular artery is traditionally described as the terminal branch of the FA, and early terminations of the FA are very rare. Lohn et al. [8] reported that among 201 facial arteries examined, only 10% terminated at the SLA and only 3% at the ILA, further highlighting the rarity of the variation presented here.

While bilateral asymmetry of the FA has been reported in the literature, the specific pattern observed in this case is exceedingly rare. Choi et al. [9] reported that 68.4% of participants demonstrated asymmetrical FA terminations, and Lohn et al. [8] found an asymmetry rate of 53%. Nevertheless, the present case is notable because the AA was supplied exclusively by the OA on the right and was entirely absent on the left. While variations in FA branching have been extensively studied, investigations focusing specifically on AA variations remain limited. Kim et al. [10] found the AA to be present as the terminal branch of the FA in 51% of 57 hemi-faces, a terminal branch of the OA in 23%, and completely absent in 26%.

Additionally, when the FA is hypoplastic, the TFA may provide compensatory supply to the AA [11]. In the present case, however, the TFA was absent bilaterally, a rare occurrence, as TFA absence has been reported in only 4% of a sample of 200 specimens [3].

The FA and its branches are derivatives of the ECA, which develops directly from the aortic sac during the fourth and fifth weeks of embryological development. The ICA follows a distinct developmental pathway, arising from the third aortic arch and the cranial aspect of the dorsal aorta [12]. The IOA, via the maxillary artery, is a branch of the ECA. The bilateral FA branch deficiencies observed in the present case likely reflect aberrant vascular development during embryogenesis, involving a combination of abnormal angiogenesis, vascular remodeling, neural crest migration, and selective regression or persistence of embryonic vascular channels [13]. Specifically, the failure of individual FA branches to develop on each side, coupled with the compensatory expansion of ECA and ICA-derived collaterals (IOA and DNA, respectively), may represent asymmetric regression of pharyngeal arch vessels that would normally give rise to these branches. Such embryological deviations can produce configurations not captured by classical anatomical classifications and may present unique surgical and procedural challenges.

Awareness of FA variations is essential for surgeons, interventional radiologists, and aesthetic practitioners, as unrecognized deviations from typical anatomy may result in serious intraoperative complications [8,9]. The asymmetries described here carry specific implications across multiple clinical domains. For example, in reconstructive surgery, the Abbe flap, which is commonly used to correct upper lip deficits from trauma, congenital deformities, or cancer, relies on axial blood supply from the ILA as its pedicle [14,15]. In the present case, the absence of a left ILA would severely compromise flap viability, potentially requiring intraoperative modification to use the contralateral ILA [16]. Similarly, surgeons should consider ILA status when planning other perioral flaps such as the vermilion flap.

The variability of the PMB is also clinically relevant. Treatment of masseter muscle hypertrophy using intramuscular botulinum toxin type A injection [17] could be affected by the presence or absence of the PMB. Pre-procedural Doppler ultrasound assessment of PMB diameter, origin, and existence may be appropriate to achieve favorable outcomes [18].

Awareness of FA variations is additionally critical in aesthetic medicine, particularly regarding the increasing use of dermal fillers. Complications, including skin necrosis and blindness from intra-arterial injection, have been documented [19]. In the present case, the right IOA dominates the arterial supply superior to the right vermilion border. Inadvertent filler injection into the IOA could propagate retrogradely via the AA and DNA toward the central retinal artery, potentially causing blindness, or toward cerebral vessels, potentially causing infarction. Both of these complications are documented risks of cosmetic filler procedures [20]. Pre-operative imaging modalities, particularly Doppler ultrasound, play a pivotal role in identifying such variants prior to intervention.

Angiographic imaging was not performed, which may have provided further insight into the vascular anatomy. This finding was limited to a single cadaver, meaning the prevalence of our variations may not reflect the broader population. However, it remains important to report such variants to avoid iatrogenesis.

## Conclusions

This case presents a rare combination of bilateral FA branch absences with compensatory flow from the IOA and DNA, a configuration not well represented in existing classification systems. The observed bilateral asymmetry, combined with TFA absence, may be overlooked without appropriate preoperative imaging. Awareness of such vascular configurations is essential for safe surgical planning, dermal filler injection protocols, and individualized pre-operative vascular mapping. Cadaveric studies remain a vital source of anatomical insight and underscore the diversity of human facial vascular anatomy. Further documentation and analysis of these variations are needed to refine existing classifications and improve patient outcomes in maxillofacial and aesthetic interventions.

## References

[1] Fakoya AO, Nessel TA, Downs BW (2024). Anatomy, Head and Neck: Facial Artery. https://www.ncbi.nlm.nih.gov/books/NBK536932/.

[2] Padur AA, Kumar N (2019). Unusual branching pattern and termination of facial artery and its clinical implications for facial operations. J Vasc Bras.

[3] Koziej M, Polak J, Wnuk J (2019). The transverse facial artery anatomy: Implications for plastic surgery procedures. PLoS One.

[4] Hong SJ, Park SE, Jo JW (2020). Variant facial artery anatomy revisited: Conventional angiography performed in 284 cases. Medicine (Baltimore).

[5] Loukas M, Hullett J, Louis RG Jr (2006). A detailed observation of variations of the facial artery, with emphasis on the superior labial artery. Surg Radiol Anat.

[6] Wang D, Xiong S, Zeng N, Wu Y (2022). Facial arterial variations in Asians: a study on computed tomographic angiography. Aesthet Surg J.

[7] Trzeciak M, Del Carmen Yika A, Glądys K (2024). The complete anatomy of the transverse facial artery: a computed tomography angiography analysis. Folia Morphol (Warsz).

[8] Lohn JW, Penn JW, Norton J, Butler PE (2011). The course and variation of the facial artery and vein: implications for facial transplantation and facial surgery. Ann Plast Surg.

[9] Choi NR, Gil YC (2025). Facial artery asymmetry and branching patterns: correlation with main trunk diameter. J Craniofac Surg.

[10] Kim YS, Choi DY, Gil YC (2014). The anatomical origin and course of the angular artery regarding its clinical implications. Dermatol Surg.

[11] Yang HJ, Gil YC, Lee HY (2010). Topographical anatomy of the transverse facial artery. Clin Anat.

[12] Bonasia S, Robert T Embryological development of the internal carotid artery. Anat of Cranial Arteries, Embryology and Variants.

[13] Udan RS, Culver JC, Dickinson ME (2013). Understanding vascular development. Wiley Interdiscip Rev Dev Biol.

[14] Bagatin M, Most SP (2002). The abbe flap in secondary cleft lip repair. Arch Facial Plast Surg.

[15] Nyame TT, Pathak A, Talbot SG (2014). The abbe flap for upper lip reconstruction. Eplasty.

[16] Schulte DL, Sherris DA, Kasperbauer JL (2001). The anatomical basis of the Abbé flap. Laryngoscope.

[17] Al-Ahmad HT, Al-Qudah MA (2006). The treatment of masseter hypertrophy with botulinum toxin type A. Saudi Med J.

[18] Wu WT, Chang KV, Chang HC (2022). Ultrasound imaging of facial vascular neural structures and relevance to aesthetic injections: a pictorial essay. Diagnostics (Basel).

[19] Schelke LW, Velthuis P, Kadouch J, Swift A (2023). Early ultrasound for diagnosis and treatment of vascular adverse events with hyaluronic acid fillers. J Am Acad Dermatol.

[20] Hong GW, Hu H, Chang K (2024). Adverse effects associated with dermal filler treatments: part II vascular complication. Diagnostics (Basel).

